# Chungsim-Yeunja-Tang decreases the inflammatory response in peripheral blood mononuclear cells from patients with cerebral infarction through an NF-κB dependent mechanism

**DOI:** 10.1186/1742-2094-7-85

**Published:** 2010-11-25

**Authors:** Hyun-Ja Jeong, In-Young Choi, Min-Ho Kim, Hyung-Min Kim, Phil-Dong Moon, Jin-Woo Hong, Soo-Hyun Kim

**Affiliations:** 1 Biochip Research Center, Hoseo University, 165, Sechul-ri, Baebang-myun, Asan, Chungnam, 336-795, Republic of Korea; 2 Department of Pharmacology, College of Oriental Medicine, Kyung Hee University, 1 Hoegi-dong, Dongdaemun-gu, Seoul, 130-701, Republic of Korea; 3 Department of Mechanical System Engineering, Chonbuk National University, Jeonju, Jeonbuk, 561-756, Republic of Korea; 4 Department of Internal Medicine, Pusan National University, School of Korean Medicine, Yangsan, Gyeongnam, 626-770, Republic of Korea; 5 Laboratory of Cytokine Immunology, Institute of Biomedical Science and Technology, College of Medicine, Konkuk University, Seoul, 143-701, Republic of Korea

## Abstract

**Background:**

Chungsim-Yeunja-Tang (CYT) has been used as a medicine for cerebral infarction (CI) patients in Korea. The objective of this study was to determine precisely the effect of CYT on CI patients using peripheral blood mononuclear cells (PBMCs).

**Methods:**

For a clinical study, 47 CI patients were identified who had taken CYT (0.01 g/kg) 3 times a day after meals for 2 weeks by oral administration. For ex vivo experiments, peripheral blood mononuclear cells (PBMCs) were isolated from CI patients. We analyzed the effect of CYT and its main components on lipopolysaccharide (LPS)-induced cytokine production and mechanism on PBMCs of CI patients by using ELISA, western blot analysis, transcription factor enzyme-linked immunoassay, and caspase assay.

**Results:**

Clinical signs of CI significantly disappeared about 2 weeks after oral administration of CYT to CI patients (*P *< 0.05). CYT and quercetin, an active compound of CYT, significantly inhibited LPS-induced interleukin (IL)-1β, IL-6, and tumor necrosis factor (TNF)-α production and expression in PBMCs. CYT and quercetin also inhibited LPS-induced nuclear translocation and DNA binding activities of nuclear factor-κB and degradation of IκBα. In addition, CYT and quercetin inhibited LPS-induced IL-32 expression and caspase-1 activation.

**Conclusion:**

These results suggest a mechanism that might explain the beneficial effect of CYT in treating CI patients. Taken together, our findings indicate that inhibition of IL-32 expression and caspase-1 activation may be a novel biomarker and potential therapeutic target in CI.

## Background

Chungsim-Yeunja-Tang (CYT), a traditional Korean medicine, has long been prescribed as a treatment for cerebral infarction (CI) to increase cerebral blood flow and to recover injured brain cells. We have previously reported that CYT regulates the serum level of cytokines in patients with acute CI [1]. However, the effects of CYT on the regulation of inflammatory cytokine production are still not completely understood. The use of herbal therapies or alternative medicines is becoming an increasingly attractive approach for the treatment of various inflammatory disorders.

Inflammatory processes are orchestrated by inflammatory cells through a complex set of chemical signals and can arise in any tissue in response to traumatic, infectious, post-ischemic, toxic, allergic, or auto-immune injury [2]. In chronic inflammatory diseases, the injury persists and leads to tissue damage [2]. During inflammation, the inflammatory region is infiltrated with mononuclear cells, producing a range of inflammatory mediators including inflammatory cytokines [3]. The expression of inflammatory cytokines is dependent on activation of a transcription factor, nuclear factor (NF)- κ B. Most commonly, NF-κB dimers are composed of *Rel A *(p65) and *NFKB1 *(p50) or *NFKB2 *(p52) subunits [4,5]. NF-κB binds to a specific consensus DNA element present in the promoter region of target genes and initiates transcription of tumor necrosis factor (TNF)-α and interleukin (IL)-6 [6,7]. NF-κB normally resides in the cytoplasm, where it is retained by association with an IκB protein (α,β, or γ), an endogenous inhibitor [5]. When activated, it translocates to the nucleus, binds to DNA, and activates genes. This activation involves the phosphorylation, ubiquitination, and degradation of IκB, leading to the nuclear migration of NF-κB [8,9]. NF-κB activation via receptor interacting protein-2 has been found to involve caspase-1 [10]. In this case, NF-κB activation by lipopolysaccharide (LPS) is attenuated in caspase-1-deficient macrophages and is inhibited by a catalytically inactive form of caspase-1 [10].

IL-32, originally named NK cell transcript 4 (NK4), is produced mainly by mitogen-activated lymphocytes, interferon-γ-activated epithelial cells, IL-12-, IL-18-, and IL-32-activated NK cells; and IL-18 gene-transfected cells [11]. Human IL-32 has six spice variants, IL-32 α, IL-32β, IL-32γ, IL-32δ, IL-32ζ, and IL-32ε [12,13]. Recombinant human IL-32γ, the most recently described inflammatory cytokine, stimulates production of IL-1β, TNF-α, and macrophage inflammatory protein-2 [14]. IL-32 stimulates the secretion of IL-1β, IL-6, IL-8, and TNF-α by activating NF-κB and p38 mitogen-activated protein kinase (MAPK) [12]. In addition, maturation of IL-1β through a caspase-1-dependent mechanism is also a property of IL-32 [14]. IL-32 production is dependent on a proinflammatory pathway involving active IL-18 induced by a caspase-1-dependt pathway [15]. These proinflammatory effects of IL-32 suggest an important role for IL-32 in inflammation.

This paper evaluates the effects of CYT and its main components on LPS-induced cytokine production and expression in peripheral blood mononuclear cells (PBMCs) of CI patients. In order to determine possible mechanisms of the inhibitory actions of CYT, we also investigated its effect on NF-κB and caspase-1 activity.

## Methods

### Materials

Ficoll-Hypaque, LPS, avidin-peroxidase, 3-[4, 5-Dimethylthiazole-2-yl]-2, 5,-diphenyl-tetrazolium bromideand (MTT), quercetin, and 2'-azino-bis (3-ethylbenzithiazoline-6-sulfonic acid) tablet substrates (ABTS) were purchased from Sigma (St. Louis, MO, USA). RPMI 1640, ampicillin, streptomycin and fetal bovine serum (FBS) were purchased from Gibco BRL (Grand Island, NY, USA). Anti-human IL-1β, TNF-α, and IL-32, biotinylated anti-human IL-1β and TNF-α, recombinant (r) human IL-1β and TNF-α, and a caspase-1 assay kit were purchased from R&D Systems (Minneapolis, MN, USA). Anti-human IL-6, biotinylated anti-human IL-6, and rhuman IL-6 were purchased from Pharmingen (San Diego, CA, USA). Recombinant IL-32 was purchased from YbdYbiotech (Seoul, Korea)

### Subjects

The subjects of this retrospective study were 47 CI patients (diagnosed by magnetic resonance imager) who had been treated with CYT in Kyung Hee Oriental Medical Center. They were hospitalized within one week of onset, treated for longer than one week, and admitted between March 1^st^, 1999 and February 28^th^, 2001. The 47 CI patients had taken CYT (0.01 g/kg) 3 times a day after meals for 2 weeks by oral administration. Thirteen volunteers also participated in PBMC isolation. Blood samples of CI patients were obtained from the Department of Neurology College of Medicine and Department of Sasang Constitutional Medicine, College of Oriental Medicine, Wonkwang University. All patients had basic tests (blood and urine tests, chest X-ray, electrocardiogram) and treatment for ischemic stroke. Informed consent was obtained from all subjects before performing these studies.

### PBMC isolation and culture

PBMCs from heparinized venous blood were isolated with Ficoll gradient centrifugation, washed three times in a phosphate-buffered saline (PBS) solution and resuspended in RPMI 1640 medium (Gibco) supplemented with 2 mM L-glutamine, 100 U/ml penicillin G, 100 μg/ml streptomycin, and 10% FBS inactivated for 30 min at 56°C. The PBMCs were adjusted to a concentration of 2 × 10^6 ^cells/ml in a 30 ml falcon tube, and 100 μl aliquots of cell suspension were placed in a four-well cell culture plate. The PBMCs were cultured for 24 h in 95% humidified air containing 5% CO_2 _(37°C). To determine whether CYT can modulate LPS-induced IL-1β, IL-6, and TNF-α production on PBMCs, the cells were pretreated with various concentrations of CYT (0.01, 0.1, and 1 mg/ml) or quercetin (0.01, 0.1, and 1 mM) for 2 h prior to LPS stimulation for 24 h, and the supernatants were collected by centrifugation and stored at -20°C.

### Preparation of CYT

The ingredients of 53.3 g of CYT included 8 g of *Nelumbo nucifera *GAERTN, 4 g of *Ophiopogon japonicus *KER-GAWL, 8 g of *Dioscoreae japonica *THUNB, 4 g of *Acorus gramineus *SOLAND, 4 g of *Scutellaria baicalensis *GEORGI, 4 g of *Ziziyphus spinosa *HU, 4 g of *Biota orientalis *ENDL, 4 g of *Euphoria longan *STEUD, 4 g of *Asparagus cochinchinensis *MERR, 4 g of *Raphanus sativus *L, 4 g of *Polygala tenuifolia *WILLD, and 1.3 g of *Chrysanthemum morifolium *RAMAT. An extract of CYT was prepared by decocting the dried mixture of herbs with boiling distilled water (100 g/l). The decoction was filtered, lyophilized, and kept at 4°C. The yield of powdered extraction is commonly about 7% (w/w). The CYT aqueous extract powder was dissolved in sterile saline (100 mg/ml) and then filtered using 0.22 μm syringe filter. 100 mg/ml CYT was diluted with saline, and we used final concentrations of 0.01, 0.1, and 1 mg/ml of CYT for the experiments. When the CYT extract was added to medium, it did not precipitate. Quercetin is a component of *Nelumbo nucifera *GAERTN (approximately 0.36-2.26%) [16], *Biota orientalis *ENDL (approximately 1.76%) [17], *Raphanus sativus *L (approximately 0.79%) [18], and *Chrysanthemum morifolium *RAMAT (approximately 0.05%) [19]. The dosage of quercetin that is found in CYT (1 mg/ml) was calculated to be about 0.02 mM. The plant materials were obtained from Oriental Medicine Hospital, Wonkwang University and identified by J.C. Joo, of the College of Oriental Medicine, Wonkwang University. The voucher specimens (voucher No. 20020427) were deposited in the herbarium in the College of Oriental Medicine at Kyung Hee University.

### MTT assay

The MTT colorimetric assay of cell survival was performed using the method of Ben Trivedi et al., [20] with minor modifications. Cell aliquots (2 × 10^5^) were seeded in microplate wells and incubated with 20 μl of a MTT solution (5 mg/ml) for 4 h at 37°C under 5% CO_2 _and 95% air. Consecutively, 250 μl of dimethyl sulfoxide (DMSO) was added to extract the MTT formazan and an automatic microplate reader measured the absorbance of each well at 540 nm.

### ELISA of IL-1β, IL-6, and TNF-α

Cytokine production was measured by a modified sandwich ELISA method, as described previously [21]. ELISA for IL-1β, IL-6, and TNF-α was carried out in duplicate in 96-well ELISA plates (Nunc) coated with each of 100 μl aliquots of mouse anti-human IL-1β, IL-6, and TNF-α monoclonal antibodies at 1.0 μg/ml in PBS at pH 7.4 and was incubated overnight at 4°C. The plates were washed in PBS containing 0.05% Tween-20 (PBST) and blocked with PBS containing 1% BSA, 5% sucrose and 0.05% NaN_3 _for 1 h. After additional washes, sample or IL-1β, IL-6, and TNF-α standards were added and incubated at 37°C for 2 h. After 2 h incubation at 37°C, the wells were washed and then each of 0.2 μg/ml of biotinylated anti-human IL-1β, IL-6, and TNF-α were added and again incubated at 37°C for 2 h. After washing the wells, avidin-peroxidase was added and plates were incubated for 45 min at 37°C. Wells were again washed and ABTS substrate (Sigma) was added. Color development was measured at 405 nm using an automated microplate ELISA reader. A standard curve was run on each assay plate using recombinant human IL-1β, IL-6, and TNF-α in serial dilutions. The inhibition percentage of cytokine production was calculated using the following equation:

%Inhibition={(c−b)−(s−b)}×100(c−b)

where c is LPS-induced cytokine production without CYT and s is LPS+CYT or quercetin-induced cytokine production (b, unstimulated cells).

### ELSIA of IL-32

IL-32 production was measured by direct ELISA method. ELISA for IL-32 was carried out in duplicate in 96-well ELISA plates coated with each of 100 μl aliquots of sample or recombinant IL-32 and was incubated overnight at 4°C. The plates were washed in PBST and blocked with PBS containing 1% BSA, 5% sucrose and 0.05% NaN_3 _for 1 h. After a 2 h incubation at 37°C, the wells were washed and then 0.2 μg/ml of anti-human IL-32 was added and the cells again incubated at 37°C for 2 h. After washing the wells, goat horse radish peroxidase conjugated antibody was added and plates were incubated for 45 min at 37°C. Wells were again washed and ABTS substrate (Sigma) was added. Color development was measured at 405 nm using an automated microplate ELISA reader. A standard curve was run on each assay plate using recombinant human IL-32 in serial dilutions.

### Reverse-transcriptase polymerase chain reaction (RT-PCR) analysis

Total RNA was isolated from PBMCs according to the manufacturer's specifications using the Easy-BLUE™RNA extraction kit (iNtRON Biotech, Taejeon, Republic of Korea). The concentration of total RNA in the final elutes was determined by spectrophotometry. Total RNA (1 μg) was heated at 65°C for 10 min and then chilled on ice. Each sample was reverse-transcribed to cDNA for 90 min at 37°C using a cDNA synthesis kit (Amersham Pharmacia Biotech, Piscataway, NJ, USA). The polymerase chain reaction (PCR) was performed with the following primers for human (h) IL-1β (5 CCG GAT CCA TGG CAC CTG TAC GAT CA 3; 5 GGG GTA CCT TAG GAA GAC ACA AAT TG 3), IL-6 (5 GAT GGA TGC TTC CAA TCT GGA T 3; 5 AGT TCT CCA TAG AGA ACA ACA TA 3), TNF-α (5 CGG GAC GTG GAG CTG GCC GAG GAG 3; 5 CAC CAG CTG GTT ATC TCT CAG CTC 3), IL-32γ (5 GTA ATG CTC CTC CCT ACT TC 3; 5 GCA AAG GTG GTG TCA GTA TC 3), and GAPDH (5 CAA AAG GGT CAT CAT CTC TG 3; 5 CCT GCT TCA CCA CCT TCT TG 3) were used to verify that equal amounts of RNA were used for reverse transcription and PCR amplification under different experimental conditions. The annealing temperature was 50°C for IL-1β, 56°C for IL-6, 60°C for TNF-α, 59°C for IL-32γ, and 62°C for GAPDH, respectively. Products were electrophoresed on a 1.5% agarose gel and visualized by staining with ethidium bromide.

### Nuclear protein extraction

Crude nuclear extract was isolated from cells. After cell activation for the times indicated, cells were washed in 1 ml of ice-cold PBS, centrifuged at 1000 × g for 5 minutes, resuspended in 400 μl of ice-cold hypotonic buffer (10 mM HEPES/KOH, 2 mM MgCl_2_, 0.1 mM EDTA, 10 mM KCl, 1 mM DTT, O.5 mM PMSF, pH 7.9), left on ice for 10 minutes, vortexed, and centrifuged at 15,000 × g for 30 s. Pelleted nuclei were gently resuspended in 50 μl of ice-cold saline buffer (50 mM HEPES/KOH, 50 mM KCl, 300 mM NaCl, 0.1 mM EDTA, 10% glycerol, 1 mM DTT, 0.5 mM PMSF, pH 7.9), left on ice for 20 min, vortexed, and centrifuged at 15,000 × g for 5 min at 4°C. Aliquots of the supernatant, which contained nuclear proteins, were frozen in liquid nitrogen and stored at -70°C. Protein was determined using a Bicinchoninic acid protein assay method (Sigma, St. Louis, MO, USA).

### Western blot analysis

Cell extracts were prepared by detergent lysis procedure. Cells (2 × 10^6 ^cells) were scraped, washed once with PBS, and resuspended in lysis buffer. Samples were vortexed for lysis for a few seconds every 15 min at 4°C for 1 h and centrifuged at 15,000 × g for 5 min at 4°C. Supernatants were assayed. Samples were heated at 95°C for 5 min, and briefly cooled on ice. Following the centrifugation at 15,000 × g for 5 min, 50 μg aliquots were resolved by 10% SDS-PAGE. Resolved proteins were electrotransferred overnight to nitrocellulose membranes in 25 mM Tris, pH 8.5, 200 mM glycerin, and 20% methanol at 25 V. Blots were blocked for at least 2 h with 1 × PBST containing 5% nonfat dry milk. Protein levels were analyzed essentially according to the manufacturer's instructions.

### Transcription factor enzyme-linked immunoassay (TF-EIA)

Avidin peroxidase was coated on a 96-well ELISA plate. The coated plate was washed with PBST and then blocked with a 3% skim milk solution. The coated plate was incubated with 1 μg/ml of 5'-biotinylated 21 single strand DNA oligonucleotide sequence for 1 h at room temperature. This sequence contains the previously described NF-κB binding motif. The sequences used here were: 5 AGT TGA GGG GAC TTT CCC AGG 3. A DNA-binding reaction was carried out in a total volume 100 μl containing 10 μg nuclear protein extract in a buffer containing 10 mM HEPES (pH 7.9), 50 mM NaCl, 5% glycerol, 1 mM EDTA, and 1 mM DTT, for 1 h at room temperature and then washed. NF-κB antibodies were then added at a 1:500 concentration in PBS containing 3% BSA for 1 h, followed by the addition of the corresponding alkaline phosphatase (AP)-coupled secondary antibody. Between each addition, the wells were extensively washed in PBST. AP activity was then detected by the addition of p-nitrophenyl phosphate (PNPP) solution (Sigma). After a 10-min incubation period, the reaction was arrested by the addition of 0.5 M H_2_SO_4_, Color intensity was detected at 405 nm using ELISA reader. AP activity was normalized to control values (unstimulated cells).

### Statistical analysis

Statistical differences between the groups were estimated using an ANOVA with a Tukey post hoc test and Mann-Whitney *U*-test. The results were considered significant at a value of *P *< 0.05.

## Results

### Effect of CYT on CI patients

Subjects included in this study were 47 patients. Table 1 shows the general characteristics of the study group. We compared the scores of NIH stroke scale (NIHSS), Modified Barthel Index (MBI), motor grade (upper limb), and motor grade (lower limb) between admission and discharge date. Discharge date scores (N = 47) of MBI, motor grade (upper limb), and motor grade (upper limb) were significantly higher than those from the admission date (*P *= 0.040, 0.030, and 0.011, respectively), but there was no significant difference in NIHSS scores (*P *= 0.086) (Table 2).

**Table 1:** Baseline characteristics of the study subjects

Characteristic	CYT Medication (N = 47)
Female sex, n (%)	30 (64)
Age, year (SD)	62.9 (13.9)
Time since stroke, day (SD)	2.1 (1.6)
Admitting day (SD)	24.7 (18.4)
Medical history	
Hypertension, n (%)	26 (55)
Diabetes Mellitus, n (%)	10 (21)
Social history	
Smoking, n(%)	14 (30)
Alcohol, n(%)	16 (34)
Motor grade (upper limb) (SD)	3.8 (1.5)
Motor grade (lower limb) (SD)	3.8 (1.4)
NIH Stroke Scale (SD)	3.6 (4.1)
Modified Barthel Index (SD)	72.4 (32.9)

CYT, Chungsim-Yeunja-Tang; SD, standard deviation; FBS, fasting blood sugar; LDL-c, low density lipoprotein cholesterol. CYT was administrated orally for 2 weeks.

**Table 2:** Changes of NIHSS, MBI, and motor grade of CYT medication group

	baseline	after 2weeks	
		
Items	Mean	Mean	P*
NIHSS	3.6 ± 4.1	2.0 ± 2.3	0.086
	
MBI	72.4 ± 32.9	86.8 ± 18.7	0.040
	
Motor grade (upper limb)	3.8 ± 1.5	4.2 ± 1.0	0.030
	
Motor grade (lower limb)	3.8 ± 1.4	4.3 ± 0.8	0.011

CYT, Chungsim-Yeunja-Tang; NIHSS, NIH stroke scale; MBI, Modified Barthel Index. N = 47.

### Effect of CYT on LPS-induced cytokine production by PBMCs

**Figure 1:**
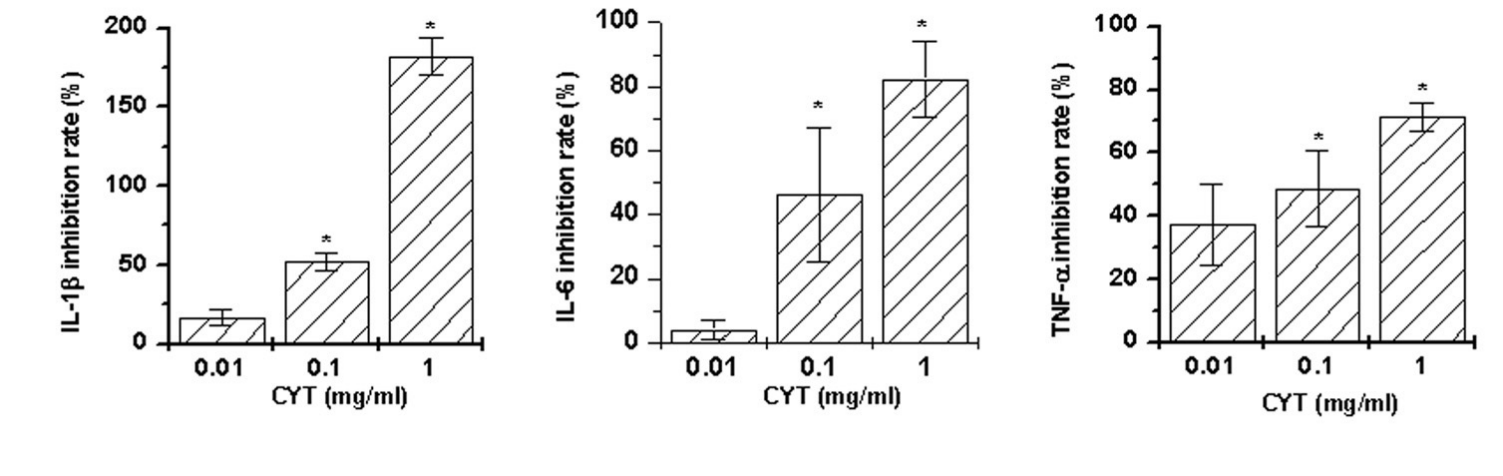
**Effect of CYT on LPS-induced cytokine production**. Peripheral blood mononuclear cells (PBMCs) (2 × 10^5^) were treated with various concentrations of Chungsim-Yeunja-Tang (CYT) for 2 h and then stimulated with LPS (10 ng/ml) for 24 h. Cytokine concentrations were measured in cell supernatants using the ELISA method. Data shown are the inhibition rate ± S.D. for 10 donors, measured in duplicate. *P < 0.05, significantly different from the unstimulated cells. **P < 0.05 compared to LPS alone.

To determine whether CYT can modulate LPS-induced IL-1β, IL-6, and TNF-α production in PBMCs, the cells were pretreated with various concentrations of CYT (0.01, 0.1, and 1 mg/ml) for 2 h prior to LPS stimulation for 24 h. Culture supernatants were assayed for IL-1β, IL-6, and TNF-α protein levels by ELISA method. LPS (10 ng/ml) increased cytokine production in the PBMCs. However, in LPS-stimulated cells, IL-1β, IL-6, and TNF-α production was decreased by treatment of CYT (about 183 ± 11.4% for IL-1β, 82.3 ± 12% for IL-6, and 71.4 ± 4.4% for TNF-α at 1 mg/ml, *P *< 0.05). CYT inhibited LPS-induced IL-1β, IL-6, and TNF-α production in a dose-dependent manner (Figure 1). Cell cytotoxicity of CYT was not observed (data not shown).

### Effect of components of CYT on LPS-induced cytokine production on PBMCs

**Figure 2:**
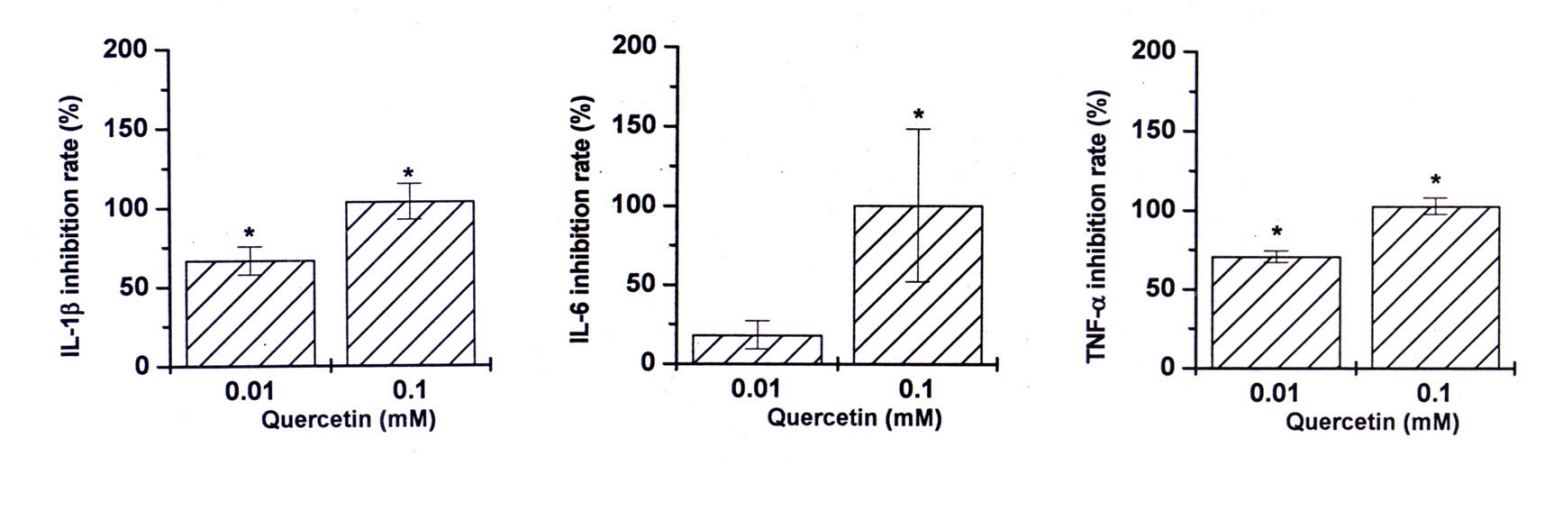
**Effect of quercetin on LPS-induced cytokine production**. PBMCs (2 × 10^5^) were treated with various concentrations of quercetin for 2 h and then stimulated with LPS (10 ng/ml) for 24 h. Cytokine concentrations were measured in cell supernatants using the ELISA method. Data shown are the inhibition rate ± S.D. for 10 donors, measured in duplicate. *P < 0.05, significantly different from the unstimulated cells. **P < 0.05 compared to LPS alone.

To determine whether quercetin, hyperoside (a component of *Nelumbo nucifera *GAERTN), or baicalein (a component of *Scutellaria baicalensis *GEORGI) can modulate LPS-induced IL-1β, IL-6, and TNF-α production on PBMCs, the cells were pretreated with various concentrations of quercetin (0.01, 0.1, and 1 mM), hyperoside (0.01, 0.1, and 1 mM), or baicalein (0.01, 0.1, and 1 mM) for 2 h prior to LPS stimulation for 24 h. Culture supernatants were assayed for IL-1β, IL-6, and TNF-α protein levels by ELISA method. Quercetin inhibited LPS-induced IL-1β, IL-6, and TNF-α production (about 103.7% for IL-1β, 100.5% for IL-6, and 102.8% for TNF-α at 0.1 mM, *P *< 0.01, Figure 2). The estimated IC_50 _values of IL-1β, IL-6, and TNF-α production were about 0.009, 0.046, and 0.007 mM, respectively. Cell cytotoxicity of quercetin at concentrations of 0.01 and 0.1 mM was not observed (data not shown) but 1 mM quercetin does show cell cytotoxicity. Hyperoside and baicalein did not affect LPS-induced inflammatory cytokine production (data not shown).

### Effects of CYT and quercetin on LPS-induced cytokine mRNA expression in PBMCs

**Figure 3:**
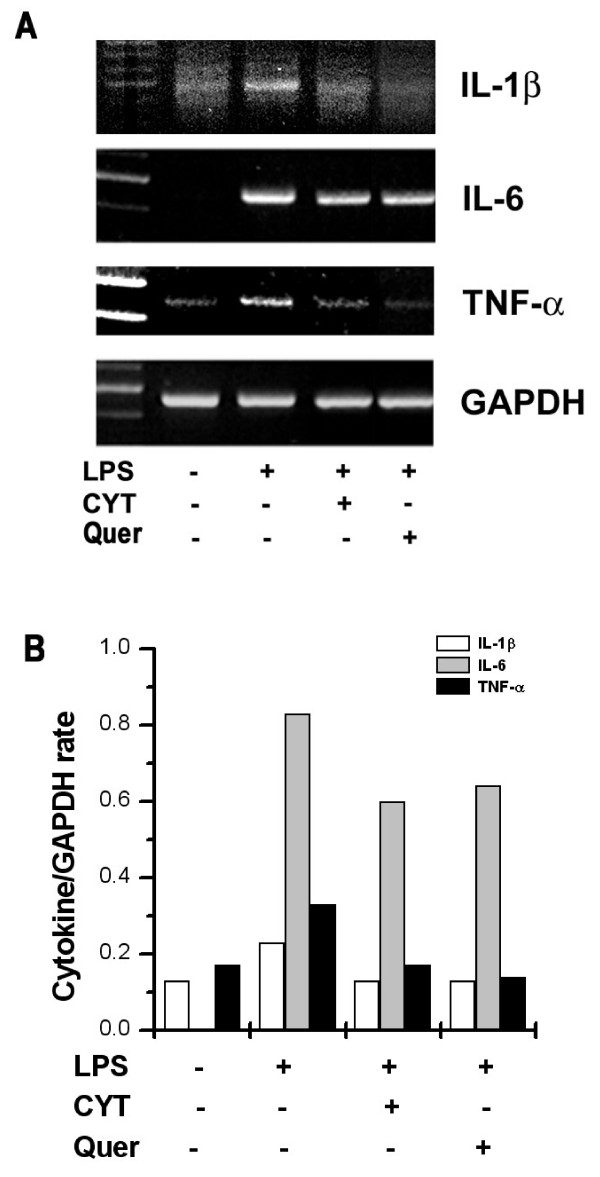
**Effects of CYT and quercetin on LPS-induced cytokine mRNA expression**. PBMCs (2 × 10^6^) were treated with CYT (1 mg/ml) and quercetin (0.1 mM) for 2 h and then stimulated with LPS (10 ng/ml) for 8 h. Messenger RNA was measured using the RT-PCR method. Results are representative of three independent experiments (A). Levels of mRNA were analyzed with densitometry (B). Quer, quercetin.

To determine whether CYT can modulate LPS-induced cytokine expression, cells were pretreated with CYT or quercetin for 2 h prior to LPS stimulation. We performed the RT-PCR analysis for IL-1β, IL-6, and TNF-αat 8 h. mRNA expression was up-regulated by LPS but the up-regulated IL-1β, IL-6, and TNF-α mRNA expression was decreased by treatment with CYT (1 mg/ml) or quercetin (0.1 mM) (Figure 3).

### Effects of CYT and quercetin on LPS-induced NF-κB activation

**Figure 4:**
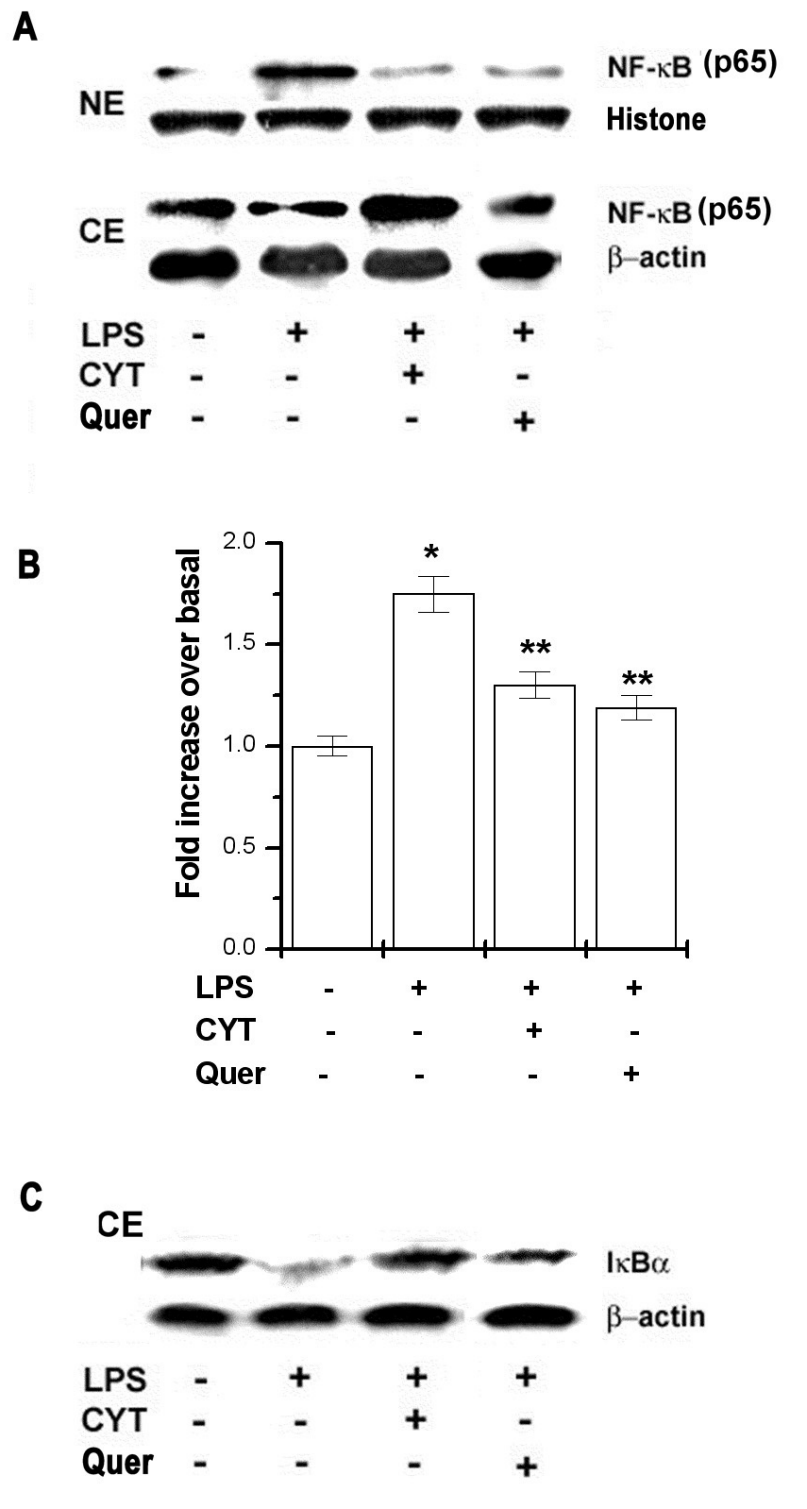
**Effects of CYT and quercetin on LPS-induced NF-κB activation and IκB degradation**. PBMCs (2 × 10^6^) were treated with CYT (1 mg/ml) and quercetin (0.1 mM) for 2 h and then stimulated with LPS (10 ng/ml) for 6 h. Nuclear and cytoplasmic protein was prepared and analyzed for NF-κB and IκBα by western blotting as described in the experimental procedures (A and C). Nuclear protein was incubated in a 96-well plate coated with an oligonucleotide containing the NF-κB binding site. Presence of NF-κB transcription complex was evaluated with an NF-κB antibody. Results are expressed as the fold increases of absorbance at 405 nm over control conditions (B). Results are representative of three independent experiments. *P < 0.05, significantly different from the unstimulated cells. **P < 0.05 compared to LPS alone.. NE, nuclear extract; CE, cytosol extract;Quer, quercetin.

The expression of inflammatory cytokines is regulated by the transcription factor, nuclear factor (NF)- κ B/*Rel *[6,7]. Expression levels of NF-κB/*Rel A *(p65) in the nucleus and cytoplasm of PBMCs was examined using western blot analysis. In LPS-stimulated cells, the expression level of NF-κB (p65) declined in the cytoplasm and concurrently increased in the nucleus. However, the expression level of NF-κB (p65) in the nucleus decreased by treatment with CYT or quercetin (Figure 4A). We also investigated the effect of CYT on LPS-induced NF-κB transcription complex. To perform these studies, we used an NF-κB TF-EIA method. This assay has the advantage of being 10 times more sensitive than an electrophoretic mobility shift assay, and it allows greater flexibility in the experimental step. As shown in Figure 4B, LPS increased DNA-binding activity for NF-κB, but this increased binding activity was decreased by treatment with CYT (1 mg/ml) or quercetin (0.1 mM). Activation of NF-κB required phosphorylation and proteolytic degradation of the inhibitory protein IκBα. To determine whether the inhibitory action of CYT was due to the effect on IκBα degradation, cytoplasmic levels of IκBα protein were examined after LPS-stimulation using a western blot analysis. CYT or quercetin also decreased degradation of IκBα (Figure 4C). Neither β-actin nor histone expression levels in the nucleus or cytosol extracts changed with either treatment.

### Effects of CYT and quercetin on LPS-induced IL-32 expression in PBMCs

**Figure 5:**
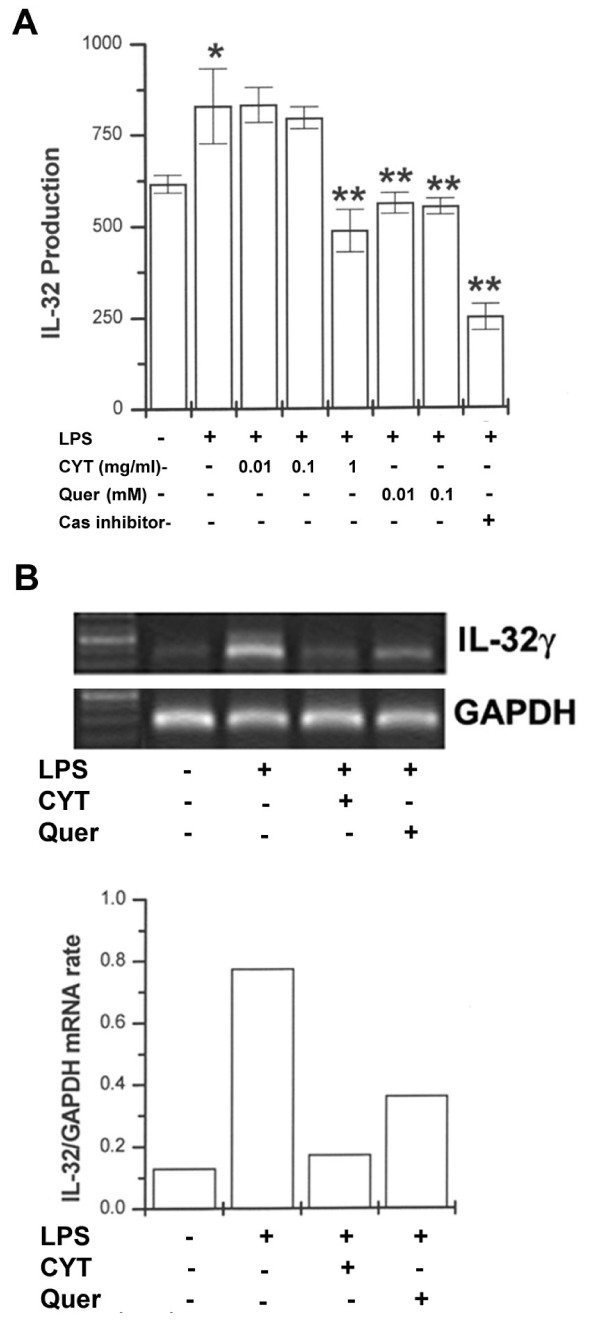
**Effects of CYT and quercetin on LPS-induced IL-32 production**. PBMCs (2 × 10^5^) were treated with CYT, quercetin, or caspase-1 inhibitor (10 μM) for 2 h and then stimulated with LPS (10 ng/ml) for 24 h. Cytokine concentrations were measured in cell supernatants using the ELISA method (A). PBMCs (2 × 10^6^) were treated with CYT (1 mg/ml) or quercetin (0.1 mM) for 2 h and then stimulated with LPS (10 ng/ml) for 8 h. Messenger RNA was measured using RT-PCR method. Results are representative of three independent experiments and band intensities correspond to levels of IL-32/GAPDH rate (B).*P < 0.05, significantly different from the unstimulated cells. **P < 0.05 compared to LPS alone. Quer, quercetin.

IL-32 stimulates the secretion of inflammatory cytokines by activating NF-κB [12]. To determine whether CYT or quercetin can modulate LPS-induced IL-32 production by PBMCs, the cells were pretreated with various concentrations of CYT (0.01, 0.1, and 1 mg/ml), quercetin (0.01 and 0.1 mM) or caspase-1 inhibitor (10 μM) for 2 h prior to LPS stimulation for 24 h. Culture supernatants were assayed for IL-32 protein levels by the ELISA method. CYT, quercetin, and caspase-1 inhibitor all significantly inhibited LPS-induced IL-32 production (Figure 5A). To determine whether CYT or quercetin can modulate LPS-induced IL-32 mRNA expression, cells were pretreated with CYT or quercetin for 2 h prior to LPS stimulation. As shown in Figure 5B, mRNA expression was up-regulated by LPS, but the up-regulation decreased with CYT (1 mg/ml) and quercetin (0.1 mM) treatments.

### Effects of CYT and quercetin on LPS-induced caspase-1 activation

**Figure 6:**
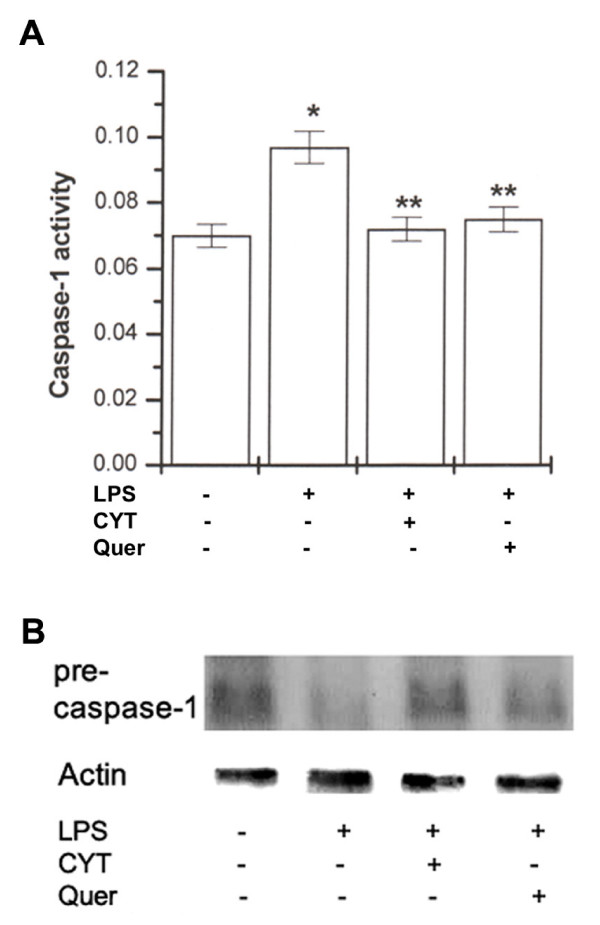
**Effects of CYT and quercetin on LPS-induced caspase-1 activation**. PBMCs (2 × 10^6^/well) were treated with CYT (1 mg/ml) or quercetin (0.1 mM) for 2 h and then stimulated with LPS (10 ng/ml) for 24 h. Caspase-1 activity was determined by a colorimetric kit using substrates (A). Caspase-1 was determined by western blot analysis (B). Data shown are the inhibition rate ± S.D. for 10 donors, measured in duplicate. *P < 0.05, significantly different from the unstimulated cells. **P < 0.05 compared to LPS alone. Quer, quercetin.

Caspase-1 is activated in a variety of inflammatory responses; caspase-1 was activated by treatment with LPS [15]. We investigated the inflammatory pathway involving caspase-1. To determine if CYT or quercetin inhibits caspase-1 activation induced by LPS, cells were exposed to LPS in the presence or absence of CYT (1 mg/ml) or quercetin (0.1 mM). Extracts prepared from PBMCs exposed to LPS contained strong caspase-1 activity compared with unstimulated cells. As shown in Figure 6A, increased caspase-1 activity was significantly inhibited by treatment with CYT or quercetin (*P *< 0.05). Caspase-1 is present in cells as an inactive zymogen and is activated by LPS treatment. The activation of caspase-1 plays an important role in NF-κB activation and IL-32 production [10,15]. Caspase-1 precursor protein was evaluated by western blot analysis. Western blot analysis indicates that LPS treatment induced degradation of caspase-1 precursor, which was inhibited by treatment CYT or quercetin (Figure 6B).

## Discussion

Herbal remedies and botanicals are widely used by humans for both preventative and curative purposes. Traditional medicinal herbs have many benefits, few (if any) side effects, and display low cytotoxicity, and so the search for the use of natural products in traditional medicine is currently attracting intense interest. In previous studies, we have investigated the effects of various traditional medicines (Yulda-Hanso-Tang, Gigukjiwhangwhan-gami, Seogak Jihwag-Tang, Jeo Dang-Tang, and Yangkyuk-Sanhwa-Tang) in patients with CI. We reported that Th2 cytokine (IL-1β, IL-4, and IL-6) levels are higher than Th1 cytokine (IL-2 and IFN-γ) levels in patients during the acute stage of CI and that traditional medicines regulate the Th1/Th2 cytokine unbalance in patients with CI [22]. As part of our continuing search for biologically active anti-inflammatory agents from oriental medicines, we investigated CYT. As described above, CYT consists of 12 different herbs. Our previous studies showed that an aqueous extract of *Asparagus cochinchinensis *inhibits the secretion of TNF-α from primary cultures of mouse astrocytes [23] and Hep G2 cells [24]. It has been reported that a fructan, Opaw-2, isolated from *Ophiopogon japonicus*, stimulates proliferation of cultured lymphocytes [25]. Extracts from *Nelumbo Nucifera *suppress cell cycle progression, cytokine gene expression, cell proliferation, and inflammatory reactions [26,27]. Zhang et al. [28] reported that an aqueous extract of *Scutellaria baicalensis *has a protective effect against acrolein-induced oxidative stress in cultured human umbilical vein endothelial cells. Some studies have suggested that wogonin, isolated from *Scutellariae Radix*, has an anti-inflammatory effect [29] and triterpenes, isolated from *Chrysanthemi Flos*, show anti-inflammatory activity against 12-O-tetradecanoylphorbol-13-acetate-induced inflammation in mice [30]. In one study, quercetin downregulated LPS-induced TNF-α and nitric oxide production [31]. In this study, CYT and quercetin inhibited LPS-induced IL-1β, IL-6, and TNF-α production in PBMCs. CYT and quercetin also increased IκB by preventing its degradation. The increased IκB inhibited the translocation of NF-κB/*Rel A *to the nucleus and the DNA binding activity of NF-κB. CYT and quercetin also inhibited IL-32 production and caspase-1 activation.

Cells activated by LPS produce cytokines that include interferons, IL-1, IL-6, IL-8, TNF-α, platelet activating factors, and procoagulant tissue factors [32]. The host response to LPS involves multiple inflammatory effector mechanisms, including cytokines [33]. IL-1 is an endogenous pyrogen, an activating factor of lymphocyte products, and is made in many cells, especially in macrophages. It activates T-cells and B-cells, causing an inflammatory response [34]. IL-6 is a pleiotropic inflammatory cytokine produced by T-cells, monocytes, and macrophages [35]. TNF-α itself promotes inflammation, leukocyte infiltration, granuloma formation and tissue fibrosis and is thought to be an initiator of cytokine-related inflammatory states by stimulating cytokine production in other types of cells [36]. Elevated levels of circulating IL-1β, IL-6, IL-8, and TNF-α have been reported previously in association with various pathological states including sepsis, rheumatoid arthritis, osteoarthritis, asthma, and CI [1,2,37-39]. In our study, CYT and quercetin inhibited IL-1β, IL-6, and TNF-α production and expression. Therefore, we suggest that CYT and quercetin inhibit inflammatory reaction by preventing the expression of inflammation-related genes. Further investigation is necessary to more precisely clarify the regulatory mechanisms of CYT involved in preventing LPS-induced inflammatory cytokine expression.

Activation of the NF-κB transcription family plays an important role in inflammation because it induces transcription of proinflammatory genes [40]. This pathway is activated via cellular stimulation, most often from signals related to pathogens or stress. New therapeutic interventions aimed at limiting the activation of NF-κB may have a beneficial effect in treating these pathological states [41]. Previously, glucocorticoids, which are frequently used in the treatment of inflammatory bowel disease and rheumatoid arthritis, were suggested to suppress NF-κB activation. Sulfasalazine and aucubin, which are potent and specific inhibitors of NF-κB, inhibit activation without preventing AP-1 binding activity [42,43]. Tang et al., reported that quercetin inhibits NF-κB activation in macrophages [44]. Our results suggest that CYT and quercetin inhibit NF-κB activation by stopping IκBα degradation. As such, CYT may modulate inflammation due to infection by preventing NF-κB activation in PBMCs.

IL-32 activates both the NF-κB and p38 MAPK pathways, which induce the gene transcription of proinflammatory cytokines, and it also activates proinflammatory caspases, such as caspase-1 [14]. IL-32-induced prostaglandin E_2 _release is important in inflammatory responses by both mouse macrophages and human blood monocytes. Following injection of human IL-32γ into knee joints of naïve mice, joint swelling with pronounced influx of inflammatory cells and cartilage damage is observed [45]. IL-32 acts in a synergistic manner with the intracellular nuclear oligomerization domain (NOD)1- and NOD2-specific muropeptides of peptidoglycans, resulting in release of IL-1β and IL-6 [14]. The synergy between IL-32 and synthetic muramyl dipeptide/NOD2 in releasing IL-6 is dependent on activation of caspase-1 and release of IL-1β [45]. Only the additive effects of IL-32 and muropeptides were observed to cause TNF-α production [45]. Production of IL-32 occurs in response to influenza A virus infection via COX-2 in the inflammatory cascade [46]. Caspase-1 is involved in inflammatory responses by causing cytokine maturation [47]. In this study, CYT and quercetin inhibited LPS-induced caspase-1 activation and IL-32 production. Caspase-1 inhibitor also prevented IL-32 production. Therefore, we suggest that CYT inhibits IL-32 production by blocking caspase-1 activation. We also found that caspase-1 and IL-32 play an important role in activating NF-κB.

## Conclusion

In conclusion, we suggest that CYT and quercetin decrease LPS-induced inflammatory cytokine production by inhibiting NF-κB and caspase-1 activation. CYT may be useful in the treatment of inflammatory diseases. However, its other components should be isolated and examined in additional studies to clarify whether they may also be effective in treating CI.

## Abbreviations

ABTS: 2'-azino-bis (3-ethylbenzithiazoline-6-sulfonic acid) tablet substrates; CI: cerebral infarction; CYT: Chungsim-Yeunja-Tang; IL: interleukin; LPS: lipopolysaccharide; NF-κB; nuclear factor-κB; MTT: 3-[4, 5-Dimethylthiazole-2-yl]-2, 5,-diphenyl-tetrazolium bromideand; PBMCs: peripheral blood mononuclear cells; TNF-α: tumor necrosis factor-α;

## Competing interests

The authors declare that they have no competing interests.

## Authors' contributions

JHJ and CIY performed the majority of the experiments and wrote the manuscript. KMH performed the ELISA. MPD and KSH provided comments on the manuscript. HJW performed sample analysis. KHM supervised the research and co-wrote the manuscript. All authors have read and approved the final version of the manuscript.
